# Transylvania University

**Published:** 1846-03

**Authors:** 


### TRANSYLVANIA UNIVERSITY.

The dean of the medical faculty of Transylvania University announces the appointment of Professor Annan, of Baltimore to the chair of Obstetrics, made vacant by the death of Professor Richardson, and that of Professor Bartlett to the chair of the Theory and Practice, which he resigned two years ago. That chair, it is stated, became vacant in consequence of the impaired health of Prof. Watson. Professor Bartlett is now in Europe, but his acceptance is spoken of in the Lexington publications as certain. For the chair to which Dr. Annan has been elected, it is reported that there were forty-nine applicants.

